# Direct and indirect effect of cannibalism and intraguild predation in the two sibling *Harmonia* ladybird beetles

**DOI:** 10.1002/ece3.6326

**Published:** 2020-06-09

**Authors:** Arash Rasekh, Naoya Osawa

**Affiliations:** ^1^ Department of Plant Protection College of Agriculture Shahid Chamran University of Ahvaz Ahvaz Iran; ^2^ Laboratory of Forest Ecology Graduate School of Agriculture Kyoto University Kyoto Japan

**Keywords:** coexistence, common species, generalist, rare species, reproductive interference, specialist

## Abstract

In this study, we focused on the direct (i.e., predation) and indirect (i.e., potential threat from coexisting with a larger individual) effects of cannibalism and intraguild predation (IGP) during larval stages of two sibling ladybird beetles. These effects play an important role in the coexistence of the generalist–common *Harmonia* *axyridis* and specialist–rare *H.* *yedoensis* (Coleoptera: Coccinellidae). Direct predation effect of cannibalism and IGP was asymmetric in the two sibling ladybird beetles; the fourth instar larvae of *H*. *axyridis* were better intraguild predators than cannibals, while the reverse was true in the larvae of *H.* *yedoensis*. Neither cannibalism nor IGP significantly affected female body weight in either species. Larval *H*. *axyridis* surviving exposure to cannibalism or IGP had a reduced number of ovarioles as adults, whereas adult *H.* *yedoensis* ovarioles were not affected. For the indirect effects, longer developmental times in males and females and a lower total number of ovarioles in females were detected in *H*. *axyridis*. In *H.* *yedoensis*, shorter developmental time of males, lighter adult weight and fewer total ovarioles in females were observed. Olfactometer choice experiments clarified that the fourth instar larvae of *H*. *axyridis* avoided the first instar conspecific larvae, while those of *H.* *yedoensis* were attracted to the odors from *H*. *axyridis* and conspecifics. Thus, *H*. *axyridis* has an avoidance mechanism only for cannibalism but not for IGP*,* whereas *H.* *yedoensis* does not have any avoidance mechanism. These different behaviors in the direct and indirect effects of cannibalism and IGP observed in the laboratory may play important roles in the coexistence of generalist–common *H*. *axyridis* and specialist–rare *H.* *yedoensis* in natural conditions, compensating for the large handicap of *H.* *yedoensis* at reproductive interference from *H*. *axyridis*.

## Graphical abstract

In this study, we focused on the direct (i.e., predation) and indirect (i.e., potential threat from coexisting a larger individual) effects of cannibalism and intraguild predation (IGP) at larval stages of two sibling ladybird beetles. These effects play an important role in the coexistence of the generalist–common *Harmonia* *axyridis* and specialist–rare *H.* *yedoensis* (Coleoptera: Coccinellidae).Different behaviors in the direct and indirect effects of cannibalism and IGP observed in the laboratory may play important roles in the coexistence of generalist–common *H*. *axyridis* and specialist–rare *H.* *yedoensis* in natural conditions, compensating for the large handicap of *H.* *yedoensis* at reproductive interference from *H*. *axyridis*.

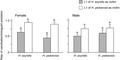

## 1 INTRODUCTION

Specialization and generalization in resource consumption are crucial issues in evolutionary ecology because they are strongly associated with mechanisms of species coexistence and phenotypic divergence (e.g., Futuyma, 2001; Mayhew, 2006). Generalists can live in different types of environments, have varied diets and can maintain large population sizes. Conversely, specialists generally have limited diets and/or require specific habitat conditions to survive, which means that they are more likely to suffer from habitat loss than common species (Schluter, 2000). Furthermore, generalists can adapt to changing environmental conditions, while the reverse is true in specialists (e.g., Townsend, Begon, & Harper, 2003).

Interspecific competition is thought to be stronger than intraspecific competition from a viewpoint of species coexistence in classical competition models (e.g., competitive Lotka‐Voltera equations; Begon, Harper, & Townsend, 1986). However, in aphidophagous guilds, the resource largely fluctuates over time and space and the resultant resource competition is normally strong for both conspecific and heterospecific individuals (e.g., Dixon, 2000; Hironori & Katsuhiro, 1997; Osawa, 2000; Ware, Yguel, & Majerus, 2009). This suggests that the circumstantial‐dependent factors (e.g., habitat heterogeneity, densities of guild members relative to prey density, and their foraging behaviors) may play an important role in the consequences of interspecific and intraspecific competition in nature.

**FIGURE 1:**
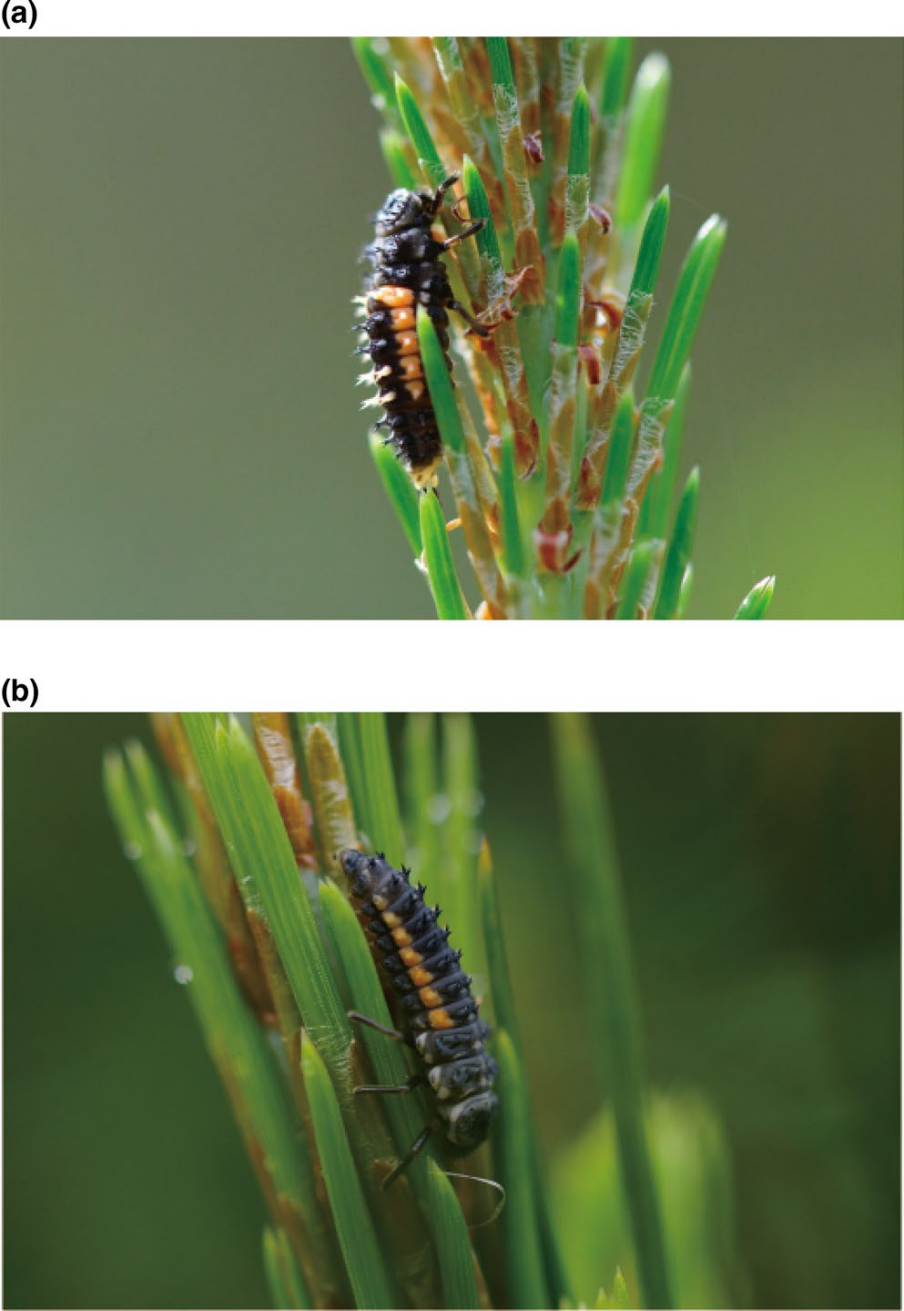
The fourth instar larva of *Harmonia* *axyridis* (a) and the third instar larva of *H.* *yedoensis* (b) on Japanese Red Pine *Pinus densiflora*

The aphidophagous ladybird beetle *Harmonia* *axyridis* (Coleoptera: Coccinellidae) is a generalist predator with a broad prey range, whereas *H*. *yedoensis* is considered a specialist predator, preying only on pine aphids (e.g., Osawa & Ohashi, 2008; Figure 1). These two sibling species (i.e., pairs of species that differ reproductively but not morphologically; Ridely, 2004) have sympatric distributions (e.g., Noriyuki & Osawa, 2012), but in Japan, can only be found simultaneously on pine trees; here, their habitats on pine trees completely overlap in time and space (Osawa & Ohashi, 2008). Interestingly, the densities of both *H*. *axyridis* and *H.* *yedoensis* are normally low on pine trees (Osawa, personal observation). *Harmonia* *axyridis* is a polyphagous ladybird that preys on a broad range of aphid species in agricultural fields, orchards, and gardens, tracking the fluctuations of resources (e.g., Koch, 2003; Osawa, 2000). Conversely, *H.* *yedoensis* mostly preys on the giant pine aphid (*Cinara pini* Linnaeus) and Thunberg's pine aphid (*Eulachnus thunbergii* Wilson; Osawa & Ohashi, 2008; Noriyuki & Osawa, 2012). The generalist and polyphagous characteristics in *H*. *axyridis*, combined with aggressive cannibalism and intraguild predation (IGP; e.g., Yasuda & Ohnuma, 1999), may play an important role in the consequences of its wide distribution as an invasive species whose colonization excludes native ladybird beetles and thus decreases aphidophagous beetle diversity (e.g., Colunga‐Garcia & Gage, 1998; Koch, 2003; Pell, Baverstock, Roy, Ware, & Majerus, 2008; Ware et al., 2009). A number of factors have contributed to the successful establishment and dominance of *H*. *axyridis* within aphidophagous guilds, including high reproductive capacity, the intensity of IGP, eurytopic nature, high resistance to natural enemies within the invaded range, and potential phenotypic plasticity (e.g., Roy & Brown, 2015; Roy et al., 2016). In addition, Honek, Martinkova, Dixon, Roy, and Pekár (2016) analyzed more than 40 years of data and suggested that climate change and habitat degradation may also be involved in the change in species composition of aphidophagous communities. Furthermore, coexistence is possible for native species and *H*. *axyridis* in invaded areas in the future due to habitat heterogeneity and the self‐regulatory population mechanisms in *H*. *axyridis* (Osawa, 2011), as well as the behavioral plasticity of related species in a theoretical model (Hently et al., 2016).

Another interesting aspect of the comparison between *H*. *axyridis* and *H.* *yedoensis* is common–rare differences (e.g., Kunin, 1997). Abundances of organisms have drawn much attention in ecology (e.g., Begon et al., 1986). Rare species are defined by low total numbers of individuals of a species, while the reverse is true for common species (Rosenzweig & Lomolino, 1997). However, the total number of individuals in a species is generally hard to clarify though *H.* *yedoensis* is rarer compared with *H*. *axyridis* in nature. In this sense, the common–rare differences in species abundance may be a consequence for the comparison of abundance in a limited area. Three sorts of traits may predispose a species towards rarity: restricted habitats, interactions with competitors or predators that severely limit their population, and a high position in the food web (Rosenzweig & Lomolino, 1997). This may remind us to consider rarity based on ecological characters in *H.* *yedoensis*.

Cannibalism and IGP are important determinants of population dynamics (e.g., Polis & Holt, 1992). In ladybird beetles, cannibalism confers some nutritional and competitive advantages for a cannibalistic larva (e.g., Dixon, 2000; Hironori & Katsuhiro, 1997; Osawa, 1992a; Pervez, Gupta, & Omkar., 2006). Cannibalism may occur in different growth stages of aphidophagous ladybird beetles, including egg, larva, prepupa, and pupa (e.g., Dixon, 2000; Osawa, 1989, 1992b; Osawa, 2002; Takahashi, 1989). It may also occur between sibling and nonsibling species (Kawauchi, 1985; Mills, 1982; Osawa, 1989; Pervez, Gupta, & Omkar., 2005), depending on a host of factors including differences in body size, population density and food availability (e.g., Michaud, 2003). The high incidence of cannibalism among ladybird beetles suggests that there may be considerable benefits of the behavior in terms of survival, providing energy, and eliminating potential competitors (e.g., Getto, Diekmann, & Roos, 2005; Martini, Garrigues, & Hemptinne, 2015; Osawa, 1992a, 1992b, 2002). By contrast, feeding on related individuals reduces the fitness of the cannibal itself (Polis, 1981). Therefore, theoretically, cannibalism in such cases should generally evolve in species with widely dispersed individuals (Lion & van Baalen, 2009).

Noriyuki, Osawa, and Nishida (2012) clarified that *H*. *axyridis* has an advantage over *H*. *yedoensis* due to asymmetric reproductive interference. This led to the conclusion that *H*. *axyridis* can force *H*. *yedoensis* to be a specialist predator that only coexists primarily in pine habitats, although the prey is relatively nutritionally poor for *H.* *yedoensis* in nature (Noriyuki & Osawa, 2012). The large amount of maternal investment and behavioral and morphological specializations of the first instars of *H*. *yedoensis*, including their long legs and ability to walk quickly, may enable them to capture pine aphids more efficiently then *H*. *axyridis*, which may play an important role in the advantage of *H*. *yedoensis* over *H*. *axyridis* in pine trees, resulting in the coexistence of these two species (Noriyuki, Osawa, & Nishida, 2011). However, theoretical models predict that coexistence between species with reproductive interference is normally difficult (e.g., Kuno, 1992), even when the effects of resource competition are included (e.g., Kishi & Nakazawa, 2013). Furthermore, reproductive interference is strongly density dependent (e.g., Takakura, 2018). This shows that the mating interference cannot fully explain the coexistence of *H*. *axyridis* and *H.* *yedoensis* because the immediate exclusion of interfered species may occur even in the pine trees, resulting in rapid exclusion of the interfered species. Therefore, their coexistence in pine trees strongly implies that there may be some kind of behavioral mechanism to resist the intense disadvantages conferred by reproductive interference. Cannibalism and IGP are considered types of direct interference between individuals, and both are important characteristics in many aphidophagous insects (e.g., Hodek & Honek, 1996). Therefore, cannibalism and IGP may function as selective driving forces for the coexistence of generalist–common *H*. *axyridis* and specialist–rare *H.* *yedoensis*.

The indirect effects of predator–prey interactions are generally defined as a reduction in prey survivorship as a consequence of a reduction in the growth rate of prey due to the presence of a predator that alters the behavior of the prey (e.g., Stamp & Bowers, 1991; Werner, Gilliam, Hall, & Mittelbach, 1983). In predator–prey relationships, predators shape prey populations by eliminating specific individuals (i.e., direct effects) and reducing modifications in prey behavior, physiology, and morphology (i.e., indirect effects), decreasing feeding and causing higher stress. This shows that resistance to both direct and indirect effects may be crucial for population growth and the consequences of interspecific competition (e.g., Jermacz & Kobak, 2017). Therefore, the direct and indirect effects of cannibalism and IGP may play important roles in governing the coexistence of *H*. *axyridis* and *H*. *yedoensis*.

In this study, we hypothesized that the direct and indirect effects of cannibalism and IGP at larval stages of the two sibling ladybird beetles might play another important role for coexistence of the generalist–specialist and common–rare relationship between *H*. *axyridis* and *H.* *yedoensis*. We examined the direct effects of cannibalism and IGP on performances closely related to fitness, that is, developmental time, body weight, and number of ovarioles, when they have access to the conspecific and heterospecific larvae at surplus food conditions. Moreover, we clarified the indirect effects of cannibalism and IGP, that is, the effects on performance when coexisting with a cannibal and intraguild predator.

## 2 MATERIALS AND METHODS

### 2.1 Insect rearing

In April 2015, we collected adults of *H*. *axyridis* (females, *n* = 10, already mated in nature and regarded as the first generation) at the Botanical Garden (ca. 1 ha.) of Kyoto University (135°47'E, 35°02'N), Kyoto, in central Japan. In May 2015, two populations of *H*. *yedoensis* (regarded as the first generation) were collected from Hieidaira, Shiga (20 females and 15 males from two egg batches; 135°83'E, 35°02'N) and the campus of Kanazawa University, Kanazawa, Ishikawa (10 females and 6 males from one egg batch; 136°70'E, 36°54'N), Japan.

In the laboratory, the adults of each species at the first generation (only individual females in *H*. *axyridis*, and a male and female pair derived from the same area, that is, Hieidaira or Kanazawa, in *H.* *yedoensis*) were separately maintained in plastic Petri dishes (9 cm in diameter by 1.5 cm high) at 25 ± 1°C, with a 16:8 hr light/dark cycle and approximately 70% relative humidity. They were provided with a surplus of artificial food every day (frozen *Ephestia kuehniella* eggs, Beneficial Insectary^®^, Redding, CA, USA). We used artificial food because (a) the food is of intermediate quality for the development of both *H*. *axyridis* and *H*. *yedoensis* (Noriyuki & Osawa, 2012) and (b) we could easily manipulate the amount of food throughout the experiment. Many egg batches were obtained from these females, and each egg batch was reared with an individual female code. Additionally, the Hieidaira and Kanazawa populations of *H.* *yedoensis* were reared differently with different area codes. The offspring were reared in plastic Petri dishes up to the adult stage under the same laboratory and dietary conditions as the females and the area codes and the sex of each egg batch was examined. The progeny of these adults, that is, the second generation, was used for experiments because we could confirm that they were *H*. *axyridis* or *H*. *yedoensis* in the larval stage (Osawa & Ohashi, 2008). Pairs of these male and female adults with different female codes were individually maintained in plastic Petri dishes with a surplus of frozen artificial food, and a clutch of eggs was transferred into Petri dishes to produce cohorts of the first and fourth instar larvae, as victims and cannibals, respectively.

The females of *H*. *axyridis* (Majerus et al., 1998; Nakamura, Ueno, & Miura, 2005) and *H*. *yedoensis* (Osawa, unpublished data) can carry male‐killing bacteria that are transmitted from mother to daughter and kill male embryos, which makes them appear to be infertile eggs. This leads to the female‐biased sex ratio observed in progeny (i.e., the female sex ratio is around 100%). Therefore, the sex of the progeny of all field‐collected *H*. *axyridis* adults and *H*. *yedoensis* egg clutches was determined prior to the test to eliminate the effects of male‐killing bacteria.

### 2.2 Food consumption and growth rate

We used newly emerged fourth instar larvae (<12 hr without sibling cannibalism) of *H*. *axyridis* and *H*. *yedoensis* in this experiment. All larvae were selected from unclustered eggs, that is, isolated eggs, in the Petri dish to prevent the selection of larva that exhibit egg cannibalism. After weighing (using a scale with 10^–4^ g accuracy) each larva (*n* = 30 for each species), they were individually introduced into Petri dishes containing a filter paper and surplus artificial food (100 mg). Then, the weight of each larva and the amount of food eaten was assessed daily until larvae pupation. Pupae were reared to adulthood to determine sex. Accordingly, the average daily and total food consumption of male and female larvae were determined. Furthermore, the growth rate of the fourth instar larvae was defined as the quantity of biomass synthesized per unit substrate assimilated. This index was calculated by dividing the wet‐weight gain of each larva based on the amount of food consumed.

### 2.3 Effects of cannibalism and IGP on cannibals and remaining victims by no‐choice experiment

All larvae used in this experiment were selected from unclustered eggs, that is, isolated eggs, in the Petri dish to prevent the selection of larva that exhibit egg cannibalism. We individually reared a larva in a Petri dish with the surplus artificial food and we individually recorded developmental period (from hatching to the fourth instar) of the larvae. Newly emerged fourth instar larvae (<12 hr without sibling cannibalism with a record of the developmental period history) were used in this experiment. After determining the weights of the larvae, they were singly introduced into a Petri dish containing a filter paper and surplus food, that is, twice the average daily consumption of the artificial food (50 mg). Based on the previous test results, the average daily consumption was determined in the fourth larval stage of each ladybird beetle. Normally *H*. *axyridis* tends to oviposite eggs before aphid density peaks (e.g., Osawa, 2000), while the oviposit timing has variations at each aphid colony in a habitat; younger and older larvae sometime simultaneously exist there (e.g., Osawa, 1992b). The same phenomenon is also observed in *H.* *yedoensis* in the field (Osawa, personal observation). Therefore, we performed cannibalism and IGP experiments to use the first instar (i.e., younger) larvae as prey and a fourth instar (i.e., older) larva as a cannibal or intraguild predator. First, 10 newly emerged first instar larvae of each species were placed in the Petri dishes. Thereafter, the fourth instar larva was introduced into the Petri dish as a cannibal and intraguild predator. The fourth instar cannibals in *H*. *axyridis* were derived from different males and females of the 10 newly emerged first instar larvae in *H*. *axyridis* and those in *H.* *yedoensis* were derived from those in *H.* *yedoensis* at the opposite area (Hieidaira or Kanazawa). Thus, between cannibal, intraguild predator and victims, there was no kin relatedness throughout all experiments. Each fourth instar larva had access to the 10 first instar conspecific or heterospecific victims under surplus food conditions. We performed the experiment under surplus food conditions to eliminate the effects of food shortage on cannibalism and IGP. We used 44 individuals (26 females and 18 males) in *H*. *axyridis* and 26 individuals (12 females and 14 males) in *H.* *yedoensis* for the experiment.

All dishes were observed daily to determine the weights of cannibal larvae and the number of prey, that is, remaining victim larvae. The artificial food was also refreshed daily, and this was continued until larvae pupation. The pupae were reared to adulthood to determine the sex of the cannibal larvae in each replicate. In the control treatment, the fourth instar larva only had access to surplus artificial food, without exposure to the first instar larvae. Body size is an important fitness component that evolves via natural selection in many animals (e.g., Roff, 1981, 1986). Furthermore, developmental time is another important factor affecting the fitness of predatory ladybird beetles (e.g., Osawa, 2002) and egg size is a function of adult weight and ovariole number (e.g., Stewart, Hemptinne, & Dixon, 1991). Hence, developmental time, adult weight, and number of ovarioles are largely involved in determining fitness. Therefore, over the course of the experiment, the developmental time (i.e., the period from hatching to prepupa) and the wet‐weight of the newly emerged adults were determined, and thereafter 3‐ to 5‐day‐old female adults were dissected to determine the total number of ovarioles in the two ovaries of each species under a stereo microscope (Carl Zeiss^®^ SV‐11 Apo). Moreover, to elucidate the effects of the existence of a large competitor (i.e., the fourth instar conspecific and heterospecific competitor) on the fitness of the first instar victims (normally the effect of existence continued 3 to 5 days as larval stage and thereafter pupal stage), prey (i.e., less than 10 individuals in number in a Petri dish) were reared with 50mg artificial food per capital to adulthood to compare the developmental time, adult weight and total number of ovarioles relative to the control larvae, that is, those that were individually reared, without conspecific and heterospecific competitors. In this experiment, we cannot exclude density effect of prey in each Petri dish, although we eliminate the effect of food shortage for prey.

### 2.4 Olfactometer choice experiment

Many studies show that olfactory cues are involved in searching behavior for larvae in ladybird beetles (Hodek & Honek, 1996), suggesting that olfactory cues also play an important role in the consequences of cannibalism and IGP. Therefore, we performed an olfactometer choice experiment to examine whether conspecific and heterospecific odor of larvae is an attractant for cannibals and intraguild predators.

The olfactometer consisted of a Y‐shaped glass tube (2.0 cm in diameter) with an entry arm (30 cm in length) and two side arms (22 cm in length, 75° apart). Each arm of the Y‐tube was connected to a glass Petri dish (9 cm diameter by 4 cm high) which functioned as an odor source with air flowing (ca. 1 m/s) through the olfactometer arms via an air pump. In each replicate, a fourth instar larvae of one species (without sexing) were introduced to the entry arm and observed until they had walked at least 15 cm up one of the arms. Each larva was used only once. Larvae that did not respond within 10 min were excluded from the replicates. The Y‐tube was cleaned with alcohol (70%) and distilled water after every replicate, and the dried Y‐tube was used for the next insect. The odor sources were switched between the left and right arms to minimize any spatial effect on choices. We assessed the responses to the emitted odor. Four conspecific and heterospecific experiments were conducted at 25 ± 1°C with 40%–50% RH as summarized below:

Treatment 1: Response of *H*. *axyridis* larvae (*n* = 14) to artificial food odor against artificial food with 10 first instar larvae of *H*. *axyridis*.

Treatment 2: Response of *H*. *axyridis* larvae (*n* = 20) to artificial food odor against artificial food with 10 first instar larvae of *H*. *yedoensis*.

Treatment 3: Response of *H*. *yedoensis* larvae (*n* = 14) to artificial food odor against artificial food with 10 first instar larvae of *H*. *axyridis*.

Treatment 4: Response of *H*. *yedoensis* larvae (*n* = 16) to artificial food odor against artificial food with 10 first instar larvae of *H*. *yedoensis*.

### 2.5 Statistical analyses

A factorial 2‐way analysis of variance test (ANOVA) was used to analyze data on food consumption and growth rate with sex and ladybird species (*H*. *axyridis* or *H*. *yedoensis*) as independent fixed factors. Data on the ratio of cannibalism and IGP were analyzed using a generalized linear model (GLM) with a binomial error distribution (Crawley, 1993), with species of cannibal and victim as independent fixed factors after data were arcsine‐transformed. A factorial 2‐way ANOVA was also used to analyze data on the fitness of larvae (cannibals and prey) with species of cannibal (*H*. *axyridis* or *H*. *yedoensis*) and species of victim (*H*. *axyridis* or *H*. *yedoensis*) as independent fixed factors (SPSS, 1998). Tukey's HSD test was used for multiple comparisons for all data (SPSS, 1998).Data from olfactometer experiments were analyzed using a chi‐square test based on the probability of each side arm (50% left, 50% right).

## 3 RESULTS

### 3.1 Food consumption and growth rate

The difference of sex and species was significant factors (*p* = .05 and *p* = .041, respectively) for growth rate, whereas sex, species, and the interaction between sex (i.e., male or female) and species (i.e., *H*. *axyridis* or *H*. *yedoensis*) were not significant factors for total and daily food consumption (Table 1).

**TABLE 1:** Results of two‐way ANOVA of the effects of sex and species of *Harmonia* *axyridis* or *H.* *yedoensis* on total and average daily food consumption and growth rate of the fourth instar larvae reared on artificial food

Source of variation	Total food consumption (g)			Average daily consumption (g)			Growth rate		
	*df*	*F*	*p*	*df*	*F*	*p*	*df*	*F*	*p*
Sex	1	0.209	.653	1	3.153	.096	1	4.536	.05
Species	1	0.742	.4	1	0.03	.865	1	2.526	.041
Sex*species	1	1.402	.252	1	3.247	.09	1	0.436	.519
Residual *df*	22			20			19		

Total and daily food consumption did not vary significantly between males and females or between species (Table 2). In terms of growth rate, *H*. *yedoensis* females exhibited significantly (*p* = .042) higher rates relative to *H*. *axyridis* females. Within *H*. *yedoensis*, the growth rate of females was significantly (*p* = .006) higher than that of males.

**TABLE 2:** Food consumption and growth rate of the fourth instar larvae of *Harmonia* *axyridis* and *H.* *yedoensis* reared on the artificial food

Dependent variable		Female (Mean ± *SE*)	Male (Mean ±*SE*)	
Total food consumption (g)	*H*. *axyridis*	0.093 ± 0.002	0.095 ± 0.009	*F* = 0.084; *df* = 1,10; *p* = .778
	*H.* *yedoensis*	0.097 ± 0.012	0.084 ± 0.004	*F* = 1.393; *df* = 1,8; *p* = .272
		*F* = 0.259; *df* = 1,10; *p* = .622	*F* = 1.411; *df* = 1,8; *p* = .269	
Average daily consumption (g)	*H*. *axyridis*	0.023 ± 0.0008	0.022 ± 0.0007	*F* = 0.542; *df* = 1,8; *p* = .482
	*H.* *yedoensis*	0.023 ± 0.0021	0.021 ± 0.0009	*F* = 1.698; *df* = 1,8; *p* = .229
		*F* = 0.001; *df* = 1,8; *p* = .983	*F* = 1.993; *df* = 1,8; *p* = .196	
Growth rate	*H*. *axyridis*	0.39 ± 0.03	0.36 ± 0.04	*F* = 0.26; *df* = 1,8; *p* = .624
	*H.* *yedoensis*	0.47 ± 0.01	0.41 ± 0.01	*F* = 14.673; *df* = 1,7; *p* = .006
		*F* = 3.667; *df* = 1,8; *p* = .042	*F* = 1.180; *df* = 1,7; *p* = .313	

### 3.2 Cannibalism and intraguild predation

**FIGURE 2:**
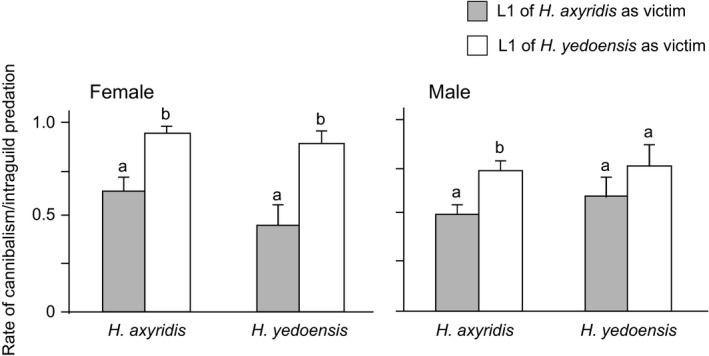
Rates of direct effects of cannibalism or intraguild predation (IGP) in *Harmonia* *axyridis* and *H*. *yedoensis* when the fourth instar cannibal larvae had access to the conspecific or heterospecific first instar victims. Vertical lines indicate S.E. Values with different small letters are significantly different (*p* < .05) in the same species of cannibal or intraguild predator when they had access to different species of victim from Tukey's HSD test

The species of the victim (*G*_1,34_ = 14.56, *p* =  .001) had a significant effect on the rate of cannibalism or IGP in females (Figure 2). The ratio of cannibalism or IGP in *H*. *axyridis* (*G*_1,24_ = 10.12, *p* = .001) and *H*. *yedoensis* (*G*_1,20_ = 6.84, *p* = .009) in females was significantly higher towards the first instar larvae of *H*. *yedoensis* than those of *H*. *axyridis*, and the same pattern was observed only in male cannibals of *H*. *axyridis* (*G*_1,16_ = 4.6, *p* = .032; Figure 2). There were no significant differences in the ratio of cannibalism or IGP of the two species for either sex (*H*. *axyridis* victim: *G*_1,18_ = 1.12, *p* = .29, *H*. *yedoensis* victim: *G*_1,18_ = 0.57, *p* = .449, in females; *H*. *axyridis* victim: *G*_1,18_ = 0.39, *p* = .553, *H*. *yedoensis* victim: *G*_1,18_ = 0.09, *p* = .765, in males) when they had access to conspecific victims (Figure 2).

### 3.3 Direct effects: Biological characteristics of cannibal and intraguild predators

The difference in species of the cannibal or intraguild predator significantly (*p* < .001 in males, *p* < .001 in females) affected the developmental time in both males and females (Table 3). Furthermore, the difference in species of the cannibal or intraguild predator, the differences in species of the victim, and their interactions were also significant for the total number of ovarioles in females (*p* < .001, *p* = .003, *p* = .025, respectively).

**TABLE 3:** Results of two‐way ANOVA of the direct effects of cannibalism and IGP on the performances

Source of variation	Developmental time (day)			Body weight of adults (g)			Total number of ovarioles		
	Female								
	*df*	*F*	*p*	*df*	*F*	*p*	*df*	*F*	*p*
Species of cannibal/intraguild predator (C)	1	48.339	<.001	1	0.037	.848	1	91.878	<.001
Species of victim (V)	2	0.945	.397	2	3.002	.059	2	6.863	.003
C* V	2	1.231	.231	2	1.745	.186	2	4.053	.025
Residual *df*	45			53			48		

	Male					
	*df*	*F*	*p*	*df*	*F*	*p*
Species of cannibal/intraguild predator (C)	1	68.635	<.001	1	0.164	.687
Species of victim (V)	2	3.159	.054	2	1.77	.17
C* V	2	1.49	.239	2	2.024	.147
Residual *df*	43			43		

**FIGURE 3:**
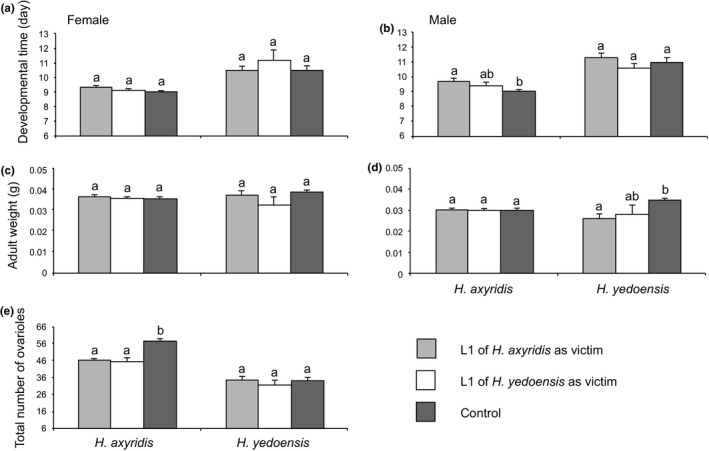
The direct effect of cannibalism and IGP in *Harmonia* *axyridis* and *H*. *yedoensis* of the fourth instar cannibals and intraguild predators on their performances when they had access to the conspecific or heterospecific first instar victims. Vertical lines indicate S.E. Values with different small letters are significantly different (*p* < .05) between species of victim for a given species of cannibal and intraguild predator from Tukey's HSD test

Developmental time was significantly longer (both in males and females) in *H*. *yedoensis* than in *H*. *axyridis* (*t*_1,43_ = 53.25, *p* < .001, in females; *t*_1,41_ = 60.65, *p* < .001, in males) but was not significantly affected by the species of the victim in either species (females: *F_2_*_,28_ = 1.93, *p* = .164 in *H*. *axyridis*; *F_2_*_,11_ = 0.57, *p* = .58, in *H*. *yedoensis*; males: *F_2_*_,20_ = 2.85, *p* = .082 in *H*. *axyridis*; *F_2_*_,17_ = 1.68, *p* = .216, in *H*. *yedoensis*; Figure 3a,b).

Cannibalism and IGP did not significantly affect the weight of adults in females of either species relative to controls (*F*_2,32_ = 0.83, *p* = .447 in *H*. *axyridis* females; *F*_2,15_ = 1.93, *p* = .18 in *H*. *yedoensis* females; *F*_2,23_ = 1.96, *p* = .175 in *H*. *axyridis* males; Figure 3c). However, a different pattern was observed in *H*. *yedoensis* males: The adult weights of specimens fed *H*. *axyridis* larvae were significantly lower than those of control specimens (*F*_2,17_ = 4.22, *p* = .033; Figure 3d).

There were significantly more ovarioles in *H*. *axyridis* than in *H*. *yedoensis* (*t*_1,11_ = 78.47, *p* < .001), and the occurrence of cannibalism (*t*_1,17_ = 36.71, *p* < .001) and IGP (*t*_1,14_ = 8.97, *p* = .01) did not change this result (Figure 3e). In *H*. *axyridis* females, cannibalism (*t*_1,17_ = 29.14, *p* < .001) and IGP (*t*_1,15_ = 6.24, *p* = .025) significantly and negatively affected the total number of ovarioles compared with controls. The total number of ovarioles did not differ significantly from controls when *H*. *yedoensis* females fed on conspecific (*t*_1,11_ = 0.91, *p* = .362) or heterospecific larvae (*t*_1,10_ = 0.002, *p* = .969)(Figure 3e).

### 3.4 Indirect effects: Biological characteristics of remaining victims

The difference of species as cannibal or intraguild predator, the difference of species as victim, and their interaction in females significantly affected their developmental time and total number of ovarioles (*p* < .001, *p* < .001, *p* < .001, respectively, for developmental time; *p* < .001, *p* < .001, *p* < .001, respectively, for total number of ovarioles; Table 4). Furthermore, the differences of species as victim significantly affected the female body weight (*p* = .03; Table 4). In males, the difference of species as victim and the interaction between the difference of species as cannibal or intraguild predator and the difference of species as victim significantly affected the developmental time (*p* < .001, *p* < .001, respectively; Table 4).

**TABLE 4:** Results of two‐way ANOVA of the indirect effects of cannibalism and IGP on the performances

Source of variation	Developmental time (day)			Body weight of adults (g)			Total number of ovarioles		
	Female								
	*df*	*F*	*p*	*df*	*F*	*p*	*df*	*F*	*p*
Species of cannibal/intraguild predator (C)	1	5.75	<.001	1	0.137	.712	1	39.6	<.001
Species of victim (V)	2	53.79	<.001	2	3.61	.03	2	38.014	<.001
C*V	2	13.78	<.001	2	2.06	.131	2	12.703	<.001
Residual *df*	157			149			145		

	Male					
	*df*	*F*	*p*	*df*	*F*	*p*
Species of cannibal/intraguild predator (C)	1	0.286	.594	1	1.178	.281
Species of victim (V)	2	11.085	<.001	2	0.398	.673
C*V	2	19.387	<.001	2	0.075	.928
Residual *df*	92			92		

**FIGURE 4:**
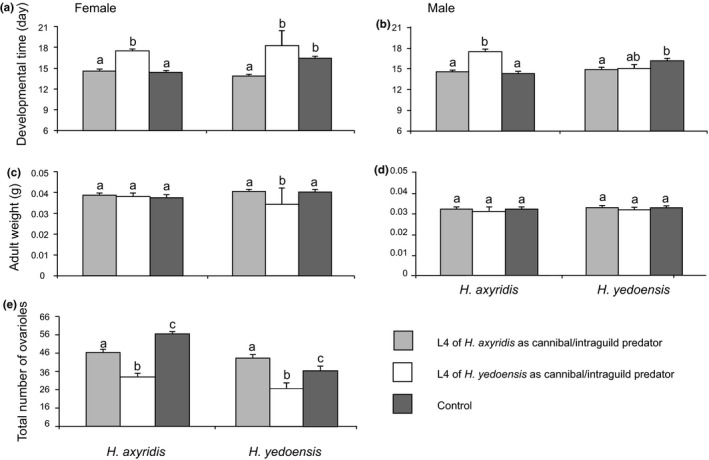
The indirect effect of cannibalism and IGP in *Harmonia* *axyridis* and *H*. *yedoensis* of remaining first instar victims on their performances after exposure to the conspecific or heterospecific fourth instar cannibal and intraguild predator. Vertical lines indicate *SE* Values with different small letters are significantly different (*p* < .05) between species of cannibal and intraguild predator for a given species of victim from Tukey's HSD test

The developmental time of males and females in *H*. *axyridis* (victim) was significantly longer when exposed to the fourth instar larvae of *H*. *yedoensis* (intraguild predator), relative to those of *H*. *axyridis* (cannibal)(females: *t*_1,74_ = 99.61, *p* < .001; males: *t*_1,40_ = 61.41, *p* < .001) and controls (females: *t*_1,19_ = 23.82, *p* < .001; males: *t*_1,12_ = 44.0, *p* < .001; Figure 4a,b). The developmental time of *H*. *yedoensis* females (victim) was significantly longer when exposed to the fourth instar larvae of *H*. *yedoensis* larvae (cannibal), relative to exposure to those of *H*. *axyridis* (intraguild predator)(*t*_1,58_ = 45.02, *p* < .001; Figure 4a). However, there were no differences between the developmental times of *H*. *axyridis* and *H*. *yedoensis* females exposed to *H*. *yedoensis* larvae (*t*_1,17_ = 1.11, *p* = .307; Figure 4a), *H*. *yedoensis* females exposed to *H*. *axyridis* larvae (*t*_1,17_ = 22.42, *p* < .001; Figure 4a), or *H*. *yedoensis* males exposed to *H*. *axyridis* (*t*_1,17_ = 6.24, *p* = .023; Figure 4b) or *H*. *yedoensis* larvae (*t*_1,19_ = 6.12, *p* = .019; Figure 4b). This resulted in shorter developmental times versus controls (Figure 3a,b). Under control conditions, the developmental time was significantly longer in *H*. *yedoensis* males and females than in *H*. *axyridis* (females: *t*_1,11_ = 46.64, *p* < .001; males: *t*_1,11_ = 42.31, *p* < .001; Figure 4a,b).

When males and females were combined, exposure to conspecific and heterospecific larvae did not significantly affect adult weight relative to controls in either species (*H*. *axyridis* females: *t*_1,63_ = 0.19, *p* = .666; *H*. *yedoensis* females: *t*_1,63_ = 0.13, *p* = .715; *H*. *axyridis* males: *t*_1,40_ = 0.011, *p* = .916; *H*. *yedoensis* males: *t*_1,28_ = 0.074, *p* = .788; Figure 4c,d). *Harmonia yedoensis* females were significantly heavier than *H*. *axyridis* females (*t*_1,115_ = 7.167, *p* = .009) when the prey was exposed to *H*. *axyrdis* larvae, although there were no significant differences between the adult weights of *H*. *axyridis* and *H*. *yedoensis* controls (*t*_1,11_ = 2.24, *p* = .163; Figure 4c). There were no significant differences between the adult weights of *H*. *yedoensis* (victim) exposed to the fourth instar larvae of *H*. *axyrdis* (intraguild predator) and *H*. *yedoensis* in the control group (*t*_1,63_ = 0.134, *p* = .715), although females under both treatments were significantly heavier relative to treatments in which the first instar larvae of *H.* *yedoensis* (remaining victims) were exposed to the fourth instar larvae of *H.* *yedoensis* (exposure to a conspecific predator larva: *t*_1,58_ = 8.63, *p* = .005, control: *t*_1,9_ = 2.6, *p* = .018; Figure 4c). There were no differences in adult weights of *H*. *axyridis* and *H*. *yedoensis* females exposed to *H*. *yedoensis* larvae (*t*_1,17_ = 0.623, *p* = .441; Figure 4c). There were no significant differences in adult weights of males of the two species (the difference between cannibalism and IGP in *H*. *axyridis*: *t*_1,56_ = 0.567, *p* = .454; *H*. *yedoensis*: *t*_1,17_ = 3.01, *p* = .101: controls: *t*_1,11_ = 0.613, *p* = .45) exposed to the conspecific or heterospecific fourth instar larvae (Figure 4d).

There were significantly more ovarioles in *H*. *axyridis* than in *H*. *yedoensis* controls (*t*_1,11_ = 261.56, *p* < .001; Figure 4e). The total number of ovarioles in *H*. *axyridis* females (victim) decreased significantly when exposed to the fourth istar larvae of *H.* *axyrdis* (cannibal) (vs. controls: *t*_1,62_ = 11.49, *p* = .001), although the negative effects of exposure to the fourth instar larvae of *H*. *yedoensis* (intraguild predator) were more intense (vs. controls: *t*_1,18_ = 41.46, *p* < .001). By contrast, the total number of ovarioles of *H*. *yedoensis* females (victim) increased significantly when exposed to the fourth instar larvae of *H*. *axyridis* (intraguild predator), relative to when they were exposed to the fourth instar larvae of *H.* *yedoensis* (*t*_1,61_ = 14.74, *p* < .001) or controls (*t*_1,56_ = 37.69, *p* < .001; Figure 4e). Furthermore, there were significantly more ovarioles in *H*. *axyridis* than in *H*. *yedoensis* (*t*_1,112_ = 7.05, *p* = .009) exposed to fourth instar larvae of *H*. *axyridis* (Figure 4e). However, there were no differences between the total number of ovarioles of *H*. *axyridis* and *H*. *yedoensis* exposed to *H*. *yedoensis* larvae (*t*_1,17_ = 1.37, *p* = .258; Figure 4e).

### 3.5 Olfactory choice tests

**FIGURE 5:**
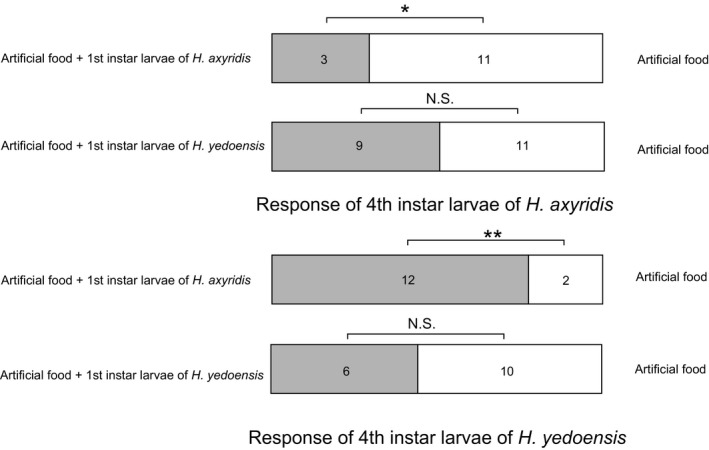
The olfactory choice response of the fourth instar larvae of *Harmonia* *axyridis* and *H*. *yedoensis* to odor from artificial food against artificial food plus 10 first instar larvae of conspecific or heterospecific individuals. The numerals on the bars indicate the number of larvae used in the experiment. *p < .05, **p < .01; N.S.: not significant (*p *> .05)

The fourth instar larvae of *H*. *axyridis* were significantly more intensely attracted to the odor emitted by the artificial food than artificial food and the first instar larvae of *H*. *axyridis* (victim) (^2^ = 4.57, *p* = .033), while there were no significant differences in the intensity of attraction to artificial food and the first instar larvae of *H.* *yedoensis* (victim) (^2^ = 0.2, *p* = .655) (Figure 5). By contrast, the odor from the artificial food and the first instar larvae of *H*. *axyridis* (victim) was more attractive for the fourth instar larvae of *H*. *yedoensis* than the odor of the artificial food (^2^ = 7.143, *p* = .008; Figure 5). However, there were no significant differences in the intensity of the attractiveness between the artificial food and the artificial food and the first instar larvae of *H.* *yedoensis* (victim) (^2^ = 1.0, *p* = .317; Figure 5).

## 4 DISCUSSION

### 4.1 Growth rate

Female *H.* *yedoensis* larvae exhibited higher growth rates than *H*. *axyridis* individuals (Tables 1, 2). In fact, *H*. *yedoensis* were larger than *H*. *axyridis* (both females and males; Figure 3). In addition, *H*. *yedoensis* females provided higher maternal investment per individual offspring than *H*. *axyridis* did; eggs of *H*. *yedoensis* were 24.91% larger than those of *H*. *axyridis* (Osawa & Ohashi, 2008). However, this tends to lead to longer hatching times for offspring, which causes delays in tracking prey aphids and high risk of cannibalism and IGP since prey density decreases rapidly during larval development (Osawa, 2002). Therefore, there is a possibility that the larger maternal investment and higher growth rate of *H*. *yedoensis* may play a role in the more intense cannibalism and IGP than that of *H*. *axyridis*, resulting in morphological and behavioral specialization in *H.* *yedoensis* to its prey.

### 4.2 The direct and indirect effects of cannibalism and IGP on life history traits

The fourth instar larvae of both ladybird beetles attacked and fed on conspecific and heterospecific victim larvae, even when they had access to sufficient food (Figure 2). Moreover, cannibalism and IGP were asymmetric in these two species; the fourth instar larvae of *H*. *axyridis* intensively chose the first instar *H*. *yedoensis* larvae over *H*. *axyridis* larvae, while the fourth instar larvae of *H*. *yedoensis* tended to choose their own species to heterospecific individuals only in females (Figure 2). These results from this laboratory experiment suggest that *H*. *axyridis* is more intensely aggressive towards other heterospecific competitors in a guild, and less so to conspecific individuals, whereas *H*. *yedoensis* is aggressive to both conspecific and heterospecific competitors. This intense characteristic in IGP and less so in cannibalism in *H*. *axyridis* may cause it to be the top predator of aphidophagous guilds (e.g., Dixon, 2000), partly resulting in its high population density, that is, as a common species in many habitats in both native and invaded areas (Koch, 2003; Roy et al., 2016). Conversely, intense cannibalism in *H*. *yedoensis* may cause low population densities, that is, rare species in natural habitats. An asymmetric relationship between cannibalism and IGP has also been reported between *H*. *axyridis* and *Coccinella septempunctata* (Hodek & Honek, 1988; Hironori & Katsuhiro, 1997; Lucas, Coderre, & Vincent, 1997). Furthermore, other differences between cannibalism and IGP have been reported (Agarwala & Dixon, 1992; Yasuda & Shinya, 1997); *Coccinella* larvae prefer to eat conspecific rather than heterospecific individuals, in contrast to *Adalia* larvae, which are better intraguild predators than cannibals (Agarwala & Dixon, 1992). There are many evidences of intense IGP (e.g., Adriaens, Branquart, & Maes, 2003; Brown, 2003; Yasuda, Evans, Kajita, Urakawa, & Takizawa, 2004; Yasuda & Ohnuma, 1999), which is considered to be related to the polyphagous feeding behavior of *H*. *axyridis*. In fact, Sloggett et al. (2011) suggested that the broad generalist diet of *H*. *axyridis*, encompassing a wide variety of aphids, additional insect prey, and other types of food (Berkvens et al., 2008; Hodek, 1996; Koch, 2003), means that this species comes into contact with, and appears to be able to tolerate, a very wide diversity of prey defensive chemistry in its diet. Michaud (2003) reported the costs of larval cannibalism with respect to eggs in terms of prolonged developmental time and reduced adult weights in *Cycloneda sanguinea*, *Olla v‐nigrum*, and *H*. *axyridis* ladybird beetles. In addition, Ware et al. (2009) showed that larvae of *H*. *axyridis* and *Adalia bipunctata* developed more slowly and have smaller adult sizes when fed a mix of conspecific eggs and aphids. We found that developmental time increased in *H*. *axyridis* males when cannibalism and IGP occurred, and the adult weight decreased in *H*. *yedoensis* males under IGP conditions (Table 3; Figure 3). The short development period is extremely important when tracking aphid density in *H*. *axyridis* (e.g., Osawa, 1992b, 2000). Furthermore, large *H*. *axyridis* males are considered superior to smaller males for mating purposes (Osawa, Kagami, Noriyuki, & Tuno, 2015; Osawa & Nishida, 1992; Ueno, Sato, & Tsuchida, 1998; Wang, Michaud, Runzhi, Fan, & Shuang, 2009). Therefore, the developmental time delay observed in *H*. *axyridis* males by cannibalism and smaller adult weights of *H*. *yedoensis* males by IGP are the detected costs in cannibalism and IGP. Generally, aphidophagous ladybird beetles may produce alkaloids that are toxic to intraguild predators that they have never encountered before over the course of their evolution (Sloggett, Haynes, & Obrycki, 2009). This suggests that IGP poses risks to the survival and development of intraguild predators. Pfennig (2000) presented the hypothesis that conspecific individuals are a better source of essential nutrients than heterospecific prey, although female fitness decreases through cannibalism. Despite these expected disadvantages, it appears that larval cannibalism and IGP can provide individual benefits for remaining larvae in terms of reduced conspecific and heterospecific competition, particularly for *H*. *yedoensis* females.

Natural enemies have a direct effect by killing prey outright, but they may also have an indirect effect on survivorship and related life history traits (e.g., Stamp & Bowers, 1991; Werner et al., 1983). Thus, we can predict that cannibalism and IGP may also have indirect effects. In this study, the developmental time increased and the total number of ovarioles decreased when males and females of *H*. *axyridis* were exposed to a predator larva of *H*. *yedoensis* relative to those exposed to conspecific larvae and controls (Table 4; Figure 4). Furthermore, the fourth instar larvae of *H*. *axyridis* tended to avoid conspecific larvae (Figure 5). Contrary to *H*. *axyridis*, the developmental times of *H*. *yedoensis* females decreased and the total number of ovarioles increased in IGP (Figure 4). The developmental times of *H*. *yedoensis* males exposed to *H*. *axyridis* larvae also decreased compared with controls (Table 4; Figure 4). The olfactory experiment confirmed that fourth instar larvae of *H*. *yedoensis* were attracted by the odor of *H*. *axyridis* larvae (Figure 5). Therefore, contrary to the negative indirect effects of IGP on *H*. *axyridis*, females of *H*. *yedoensis* did not pay any cost in terms of fitness traits (i.e., number of ovarioles) due to IGP (Figure 4). This result observed in indirect effects in a laboratory setting may confirm the evolutionary adaptation of *H.* *yedoensis* females to the performances of IGP, although negative effects of cannibalism were observed.

In sibling cannibalism of *H*. *axyridis* at hatching, that is, newly hatched larvae eating sibling eggs, sexual differences were detected as a consequence to life history traits: 4.24% and 1.22% decreases in developmental time and 2.32% and 1.05% increases in body size in males and females, respectively (Osawa, 2002). However, we did not observe any significant differences in the developmental times and adult body weights of females with cannibalism and IGP and females whose entire larval stages were exposed to conspecific and heterospecific predators, *H*. *axyridis* and *H*. *yedoensis* (Tables 3, 4; Figures 3, 4), showing that the direct and indirect effects of cannibalism and IGP do not affect female body weight, per se. However, females experiencing the direct and indirect effects of cannibalism and IGP have fewer ovarioles than the controls in *H*. *axyridis*. The number of ovarioles and body size are generally the main determinants for egg size in ladybird beetles—species with fewer ovarioles lay larger eggs than similarly sized species with many ovarioles (e.g., Stewart et al., 1991)—showing that individuals with fewer ovarioles lay larger eggs than those with many ovarioles among individuals of the same size from a given species. Furthermore, Hemptinne, Dixon, and Coffin (1992) suggested that gravid female aphidophagous ladybird beetles mainly use the presence of conspecific larvae to assess the potential of an aphid colony for supporting the development of their offspring. Therefore, *H*. *axyridis* females may modify fitness‐related characteristics, laying fewer, larger eggs (i.e., larger maternal investment by the female) in relation to the environmental conditions with intense cannibalism and IGP. On the contrary, no effect was detected for number of ovarioles in *H.* *yedoensis* females by the direct effects of cannibalism and IGP, indicating that the maternal investment in the egg, that is, egg size, is not changed by cannibalism and IGP in *H.* *yedoensis*. However, the number of ovarioles with indirect IGP was larger than that in controls in *H.* *yedoensis*, whereas the number of ovarioles decreased in response to indirect cannibalism, indicating that plasticity of the maternal investment in the egg is intense as a result of indirect effects of cannibalism and IGP in *H.* *yedoensis*.

This study showed that the rate of cannibalism and IGP toward the victim of *H.* *yedoensis* was more intense than toward *H*. *axyridis* (Figure 2), whereas preference of odor of the fourth instar *H.* *yedoensis* larvae towards the first instar larvae was not intense (Figure 5). This shows that the high rate of cannibalism and IGP in *H*. *axyridis* and *H.* *yedoensis* towards the victim of *H.* *yedoensis* (Figure 2) was not caused by the odor from *H.* *yedoensis* victims. Many authors have reported that neither optic nor olfactory orientation operate in prey searching behavior, whereas the reverse is also true in many studies (cf. Hodek & Honek, 1996). Storch (1976) clarified that the prolegs and possibly the head and mouthparts are more important than the stemmata for detecting prey in *C. transversoguttata* larvae. However, no study clarified the effects of larval existence on prey searching behaviors of larvae in ladybird beetles. It is known that morphological and behavioral analyses showed that hatchlings of *H.* *yedoensis* locomoted; they had longer legs and a larger head capsule size and could walk faster than *H*. *axyridis* (Noriyuki et al., 2011). Therefore, this discrepancy may partly be explained by the two circumstantial evidences that (a) the encounter rate is higher between the fourth instar larvae of *H*. *axyridis* and *H.* *yedoensis* and the first instar larvae of *H.* *yedoensis* at the Petri dishes, and (b) lower preference of the fourth instar *H*. *axyridis* larvae for the first instar larvae of their own species and no preference of the fourth instar *H.* *yedoensis* larvae between the first instar larvae of *H*. *axyridis* and *H.* *yedoensis* (Figure 5).

In this study, we were limited laboratory experimental approaches to clarify the direct and indirect effects of cannibalism and IGP in the two sibling *Harmonia* species. As we cannot observe cannibalism and IGP behavior with accuracy in the field, there is scant chance of the direct observations. Additionally, it is very difficult to clarify the morphological distinctions between the two species. However, we must note that the results in this study were obtained in strictly controlled laboratory conditions and many factors (e.g., size and structure of a habitat, odor from preys, and conspecific and heterospecific competitors, and behavioral and size effects in larvae for cannibalism and IGP at each species) may be simultaneously involved in nature.

## CONFLICT OF INTEREST

The authors have no conflict of interest associate with this manuscript.

## AUTHORS' CONTRIBUTIONS

NO: Idea originally formulate. RA: Experiment performance; data collection; and data analysis. RA and NO: Experiment conception; experiment design; and manuscript writing. All authors contributed critically to the drafts and gave final approval for publication.

## Data Availability

All the data are available from the Dryad Digital Repository: https://doi:10.5061/dryad.547d7wm59
